# Induction of Autophagic Pathways in Alzheimer’s Disease via SARS‐CoV‐2 Vaccination: A Deep Learning Analysis of Neurodegenerative Modulation

**DOI:** 10.1002/alz.095435

**Published:** 2025-01-09

**Authors:** Rifaldy Fajar, Elfiany Elfiany, Sahnaz V Putri, Prihantini Prihantini

**Affiliations:** ^1^ Yogyakarta State University, Sleman, Special Region of Yogyakarta Indonesia; ^2^ BLK General Hospital, Bulukumba, South Sulawesi Indonesia; ^3^ International University Semen Indonesia, Gresik, East Java Indonesia; ^4^ Bandung Institute of Technology, Bandung, West Java Indonesia

## Abstract

**Background:**

Emerging research indicates that autophagy, a cellular degradation process, may be triggered by certain immune responses, including those by vaccines. This study aims to examine whether the SARS‐CoV‐2 vaccines, known to induce robust immune activation, can trigger autophagic pathways that facilitate the degradation of amyloid‐beta (Aβ), a pathological feature of Alzheimer’s disease (AD). By applying deep learning techniques to analyze complex immunological and neurological data, this study explores a potentially innovative therapeutic strategy for AD.

**Method:**

In this study, we conducted a focused cohort involving 1,200 early to mid‐stage Alzheimer’s disease (AD) patients who received the SARS‐CoV‐2 vaccine, along with 1,200 matched controls who did not receive vaccination. Over a period of 24 months, we gathered and analyzed extensive datasets, which included genomic information related to autophagy‐related genes, longitudinal blood biomarker profiles emphasizing autophagy and amyloid‐beta (Aβ) markers, and detailed brain imaging data for quantifying Aβ deposition and brain morphology changes. Our analysis utilized deep learning models, specifically designed to process sequential and spatial data, to identify patterns that correlate vaccine response with changes in autophagic activity and Aβ levels.

**Result:**

Preliminary analysis revealed that vaccinated AD patients exhibited a 24% increase in markers of autophagic activity (95% CI: 20%‐28%, p<0.001) and a corresponding 22% reduction in soluble Aβ levels (95% CI: 18%‐26%, p<0.001) compared to baseline and control groups. Cognitive assessments showed a deceleration in cognitive decline in the vaccinated group, with a statistically significant difference in the annual decrease in MMSE scores by 1.1 points (95% CI: 0.7‐1.5, p<0.001) relative to unvaccinated patients. Deep learning models identified a predictive interaction between specific autophagy‐related genetic variants and vaccine response, indicating higher efficacy in certain subgroups.

**Conclusion:**

The study suggests that SARS‐CoV‐2 vaccination may improve autophagy in Alzheimer’s brains, aiding in amyloid‐beta breakdown. This has implications for treating Alzheimer’s and potentially other neurological conditions with vaccines or immunotherapies targeting autophagy.

